# The Influence of Teachers’ Self-Efficacy and School Leaders’ Transformational Leadership Practices on Teachers’ Innovative Behaviour

**DOI:** 10.3390/ijerph18126423

**Published:** 2021-06-14

**Authors:** Mohammed Afandi Zainal, Mohd Effendi Ewan Mohd Matore

**Affiliations:** Research Centre of Education Leadership and Policy, Faculty of Education, Universiti Kebangsaan Malaysia, Bangi 43600, Selangor, Malaysia; afan.zainal@gmail.com

**Keywords:** innovative behaviour, self-efficacy, transformational leadership, school administrators, teachers

## Abstract

Background: The current rapid development demands educators to possess innovative behaviour. Presently, in the environment of rapid technological growth and borderless global communication, teachers with innovative behaviour are capable of facing each painful challenge that confronts the education system; therefore, it is essential to understand the factors influencing the innovative behaviour of teachers. Purpose: To identify the influence of teachers’ self-efficacy and school administrators’ transformational leadership practices on teachers’ innovative behaviour. Method: A quantitative approach using a cross-sectional survey design with a sample of 1415 teachers from four states in Malaysia, and the data were statistically analysed using SPSS^®^ version 26.0 for Windows™ (IBM Corporation, New York, NY, USA). Result: Multiple Regression Analysis found that teachers’ self-efficacy and school administrators’ transformational leadership practices both had a significant influence on teachers’ innovative behaviour by contributing 47.0% of the variance in teachers’ innovative behaviour. Conclusion: The findings suggested that teachers’ self-efficacy and school administrators’ transformational leadership practices both play a role in influencing teachers’ innovative behaviour. Therefore, the stakeholders need to consider the aspects of self-efficacy and transformational leadership practices of school administrators in drafting policies and related programmes to improve teachers’ innovative behaviour.

## 1. Introduction

The current global environment, which is becoming more challenging and competitive, has made innovation and innovating capability important determinants for keeping pace with the times for any individual, group, community, organisation, or country [1]. To avoid being overtaken and left behind due to current changes, individuals or organisations therefore need to innovate continually. Innovation, commonly meant as a change, renewal, improvement, and opportunity creation, is deemed crucial for survival, success, growth, and excellence in order to move towards something better. Most countries worldwide have recognised the undeniable importance of innovation and its desirable impact; however, realising or creating innovation is not as easy as perceived by some people. Innovating is not complicated, but in ensuring the success of innovation, basic elements such as how innovation can occur should be understood first. As a matter of fact, innovation does not occur naturally [2]. Theoretically, whether or not innovation can occur in an organisation depends on the individuals in the organisation [3,4]. According to [5,6], individuals or human resources in an organisation are the most important aspect in ensuring the success of an innovation. Without individuals in the organisation, it would be impossible for innovation to take place because it requires an insistent awareness, desire, and attitudinal change in individuals [7,8]. As such, individuals need to possess the attitude and desire to transform the existing matters into something better even when the current situation is already excellent in order to support the growth and creation of innovation with innovative behaviour [6,9,10,11]. This type of behaviour has a specific term known as Innovative Behaviour or Innovative Work Behaviour.

### Research Background

Innovative behaviour is a group of behaviours with a series of different behavioural activities directed towards the creation of innovation, which involves exploring opportunities, generating ideas, promoting ideas, and realising ideas [12,13]. Specifically, innovative behaviour refers to individuals’ behaviours and actions that are intentionally directed towards the creation, development, or application of new things such as a product, technology, service, or a change in procedure or work process to improve the existing work in order to increase the efficiency and effectiveness of an organisation [12,14,15,16,17,18].

In the education field, the innovative behaviour that should be prioritised and focused on is innovative behaviour among teachers because teachers are not only the largest unit in this field, but they are also the main drivers of the education system. Besides, in the environment of rapid technological growth and borderless global communication today, teachers with innovative behaviour are seen as capable of facing each painful challenge that confronts the education system [19,20,21]. Thus, ensuring that the education system remains relevant and in line with the moving times is one of the main challenges to be faced in a rapidly changing open environment and, as such, the education system cannot be static, but rather be dynamic and continually changes to fulfil the aspirations and needs to be at par and relevant with current developments. Additionally, the creation of innovation in the education field depends on teachers’ innovative behaviour [22,23,24,25]; therefore, it is important to equip teachers with this type of behaviour. 

In Malaysia, teachers’ innovative behaviour is deemed a lingering issue. Based on the surveys conducted in the past few years, teachers’ innovative behaviour has not reached an outstanding level in this country and the findings of previous studies also showed inconsistency in teachers’ innovative behaviour in Malaysia between 2012 and 2019, besides remaining at low and average levels without any significant improvement [26,27,28,29,30]. This phenomenon is a serious issue that requires attention because a prolonged lack of innovative behaviour among teachers is detrimental to any education system in the long run, either directly or indirectly [2,31,32] and one of the significant direct impacts that can be predicted is the creation of innovation [33,34]. 

In view of this issue, immediate action and further research, therefore, needs to be taken to ensure that such an issue will not continue to plague the education system. In this context, efforts to elevate teachers’ innovative behaviour, which is currently unsatisfactory, should be prioritised. One of the efforts that can be taken to better address this critical issue is by enhancing knowledge regarding the factors that contribute to teachers’ innovative behaviour in the context of Malaysia in order to provide a clearer picture and understanding of teachers’ innovative behaviour. Consequently, such understanding will help improve the level of innovative behaviour among teachers by enabling further exploration of the progressive development of innovative behaviour in the future.

## 2. Literature Review and Hypothesis Development

This study aims to determine the contribution of several factors to teachers’ innovative behaviour. Accordingly, a basis of the proposed theory by [10] on individual innovative behaviour was chosen to underlie this study, which presumes individual innovative behaviour as the outcome of several interacting components and factors such as demographics, individuals or personals, and organisations. With the multitude of factors believed to influence innovative behaviour, it was arduous to determine the most dominant and influential factors to be examined in this study; however, this study only selected the factors of teachers’ self-efficacy and school administrators’ transformational leadership practices, meticulously based on several considerations such as the literature review, underpinning theories, and related empirical evidence. 

An early review of the literature has demonstrated the potential of self-efficacy and transformational leadership style in contributing to innovative behaviour; however, little work has been carried out on the transformational leadership style and teachers’ self-efficacy in recent decades, while previous works have not comprehensively considered these factors towards influencing teachers’ innovative behaviour. Based on the Systematic Literature Review (SLR) along with its comparisons to other works [35], the pattern of the studies shows a strong justification for both factors of transformational leadership and self-efficacy in influencing teachers’ innovative behaviour; however, there is a lack of comprehensive identifications of the factors influencing this behaviour. While the study clearly mentions how its research focus advances knowledge in the field, the study by [35] also aims to systematically identify the factors that influence innovative behaviour among teachers for the current ten-year study period. Evidently, the study identified a total of 46 factors from three groups, namely demographics, individuals or personals, and organisations; hence, it can be concluded that innovative behaviour among teachers is not merely influenced by a single factor. Like other human behaviours, many factors and variables can likewise influence innovative behaviour and, as such, the two factors found with the highest influence on teachers’ innovative behaviour are leadership and self-efficacy. Although this SLR study is the biggest driver of the rationale for choosing self-efficacy and transformational leadership practices, detailed empirical studies are necessary to see whether or not these two factors influence teachers’ innovative behaviour.

In terms of the theoretical consideration, the connection between teachers’ self-efficacy and innovative behaviour can be explained using Bandura’s Social Cognitive Theory [36] about human behaviour as a dynamic interaction of personal, behavioural, and environmental influences. Looking deeper into Bandura’s work, self-efficacy may influence human behaviour through numerous different processes. Firstly, self-efficacy influences how people undertake tasks so that they can perform tasks that they believe can be completed successfully. Next, self-efficacy influences the effort that people will be inclined to employ on a task including their perseverance. Lastly, self-efficacy influences people’s effective responses to approaching tasks, which in turn influences the degree of successful completion of the task. In simpler words, when the individuals believe that they can generate the desired result through their actions, they are more likely to act and achieve them [37]. This is because the level of self-efficacy influences motivation, effort, perseverance in the face of difficulty, emotional stability, and stress levels [38]. For this reason, a high self-efficacy level should increase the determination of individuals and allow them to perform better in their desire for success [39]. Additionally, this is highly related to innovative behaviour since innovation activity and innovative behaviour are known as difficult activity due to the high risk of failure and uncertainty [40,41,42,43]. Therefore, to engage in activities related to innovation, individuals require courage and the willingness to face possible uncertainties and risks. Nonetheless, through self-efficacy, this is seen to be passable without any difficulties because individuals with high self-efficacy are evidently more willing and courageous to take risks [42,44]. In fact, they believe that they have the ability to perform their duties according to set standards [36,45]. Past studies have also found that individuals with high self-efficacy are more creative [46] and more inclined to perform challenging tasks [47], which is in line with the need for innovation. Other than that, [48] stated that teachers’ self-efficacy is a good predictor for various types of teacher behaviours, which relevantly explains teachers’ behaviour or activities. Thus, we believe that self-efficacy is relevant in explaining innovative behaviour among teachers.

To explain the theoretical connection between transformational leadership and innovative behaviour, the seminal theory about transformational leadership from [49] should be referred to. This leadership style, according to [50], is adaptively ideal, with the involvement of extraordinary forms of influence of leaders in terms of emotions, values, ethics, standards, and long-term goals that move subordinates to perform better than expected through the transformation of subordinate attitudes, beliefs, values, and behaviours [51,52]. Besides, [49] theorised that transformational leaders exhibit four types of behaviours that include idealised influence, intellectual stimulation, individualised consideration, and inspirational motivation. In relation to innovative behaviour, this leadership style may lead to increased innovation through increased creativity among subordinates [52,53,54]. According to [55], one of the behaviours of a transformational leader is that the individualised consideration of the leader could encourage subordinates to reciprocate with greater creativity and innovativeness in line with the social exchange perspective [56]. Another dimension in transformational leadership is also associated with innovative behaviour called intellectual stimulation through which leaders encourage subordinates to think more creatively, differently, and boldly to take risks for transformation, changes, and even problem-solving [57,58]. Hence, we believe that transformational leadership is suitable for supporting the notion of innovative behaviour among teachers.

Based on early considerations, research on the factors influencing teachers’ innovative behaviour remains limited and has not gained thorough attention in Malaysia [28,59]. The vast opportunities and areas to be explored also call for a study that examines the influence of teachers’ self-efficacy and transformational leadership practices on teachers’ innovative behaviour; therefore, the following hypothesis was proposed in this study:

H1: *Teachers’ self-efficacy and school leaders’ transformational leadership practices have a positive relationship with teachers’ innovative behaviour.*

## 3. Materials and Methods

### 3.1. Research Design and Participants

This study used a quantitative approach using the survey method to examine the influence of the independent variables (teachers’ self-efficacy and school leaders’ transformational leadership practices) on the dependent variable (teachers’ innovative behaviour). School leaders in this context refer to the principals/headmasters/headmistresses. 

The sample size was estimated using the G*Power version 3.1.9.4 statistical software (Heinrich Hein University, Düsseldorf, Germany), where the alpha value was 0.05, the power was 0.9, and the effect size was set at 0.02. The calculated results further estimated a minimum sample size of 1073 respondents; however, after a 40% possible loss rate (respondents refusing to participate or withdrawing) was considered [60], the actual sample size required for this study was 1500 respondents. In general, the population of this study refers to teachers who are currently serving in national (primary) schools and government-aided national (secondary) secondary schools in Peninsular Malaysia. Since the population size is large and involves a large geographical area, this study combines several sampling techniques such as cluster sampling, simple random sampling, and stratified sampling to select samples from the research population. There were no invalid questionnaires; hence, no single questionnaire was deleted. Finally, a total of 1415 questionnaires were collected, yielding a return rate of 94.33%. 

### 3.2. Survey Instrument

This study used a structured questionnaire for data collection that includes four parts, namely the respondents’ sociodemographic characteristics, teachers’ innovative behaviour scale, teachers’ efficacy scale, and school leaders’ transformational leadership scale, which were adapted from several sources. The teachers’ efficacy scale was adapted from the studies by [61,62,63], while the teachers’ innovative behaviour scale was based on the innovative behaviour measurement by [12,13], interwoven and modified based on previous studies [64,65,66] for suitability in the local context. Finally, the school leaders’ transformational leadership scale encompassed a process of adaptation and modification of the instrument used by [67,68]. These three instruments are explained in Section 3.2.3, Section 3.2.4 and Section 3.2.5, and the list of adapted items are outlined in Appendix A.

#### 3.2.1. Validity and Reliability

Various methods can be used to determine the validity and reliability of an instrument; however, this study only used several specific methods such as a content validity approach through expert consensus (validity) as well as internal consistency and item reliability through Rasch Measurement Model (reliability). Specifically, content validity through expert consensus in this study was conducted using the Content Validity Ratio (CVR) method, which was introduced around 1975 by Charles Lawshe. Using this method, a mutual agreement among experts (evaluators) was measured based on the level of importance and suitability of each item in the instrument. Prior to content validity, the initial number of the adapted items used in the instrument was identified to comprise 87 items that consist of teachers’ self-efficacy with 28 items, teachers’ innovative behaviour with 34 items, and school leaders’ transformational leadership practices with 25 items. 

Based on the expert panel’s evaluation as shown in Table 1, a total of 14 items had recorded CVR values below the required cut off level, while the rest of the items for measuring each construct were certified. Specifically, seven items for innovative behaviour did not reach the required cut off level, while teachers’ self-efficacy and transformational leadership recorded four and three items, respectively. The experts further raised two main issues of recurring items (9 items) and item clarity (5 items); however, based on their comments and suggestions, the researchers could enhance the quality of the instrument by improving some items with clarity issues and dropping repetitive items. After conducting some review, the researchers decided to drop 4 items for innovative behaviour and self-efficacy, respectively; however, no items were dropped for transformational leadership. In summary, out of the total 87 items initially provided for content validity through expert consensus, 8 items had been dropped, and only 79 items were used for the pilot study.

After the expert validation process, the survey instrument conclusively includes 79 items, and the data obtained from the pilot study was analysed using the Rasch measurement model to identify problematic items and to obtain the reliability values of the instrument. The pilot study was conducted in one of the national primary schools and national secondary schools in one of the states in Malaysia. To perform the Rasch Measurement Model analysis on the pilot study data, the number of samples was determined based on the sample determination table by [69] and the minimum sample size required was 100 respondents for a confidence level of 95% for ±0.5 logit. A total of 109 samples, specifically 59 teachers from the national primary school and 50 individuals from the national secondary school, were involved in this pilot study over one week. Several aspects of the Rasch model considered at this stage include (a) item fit (Table 2, Table 3 and Table 4), (b) item polarity (Table 5), (c) unidimensionality (Table 5), and (d) local independence (Table 6).

As shown in Table 2, Table 3 and Table 4 for item fit, the range of *infit* Mean Square (MNSQ) was within 0.80 to 1.28, whereas the *outfit* MNSQ range was within 0.79 to 1.26. Meanwhile, for *z*-std, the *infit* was ranging from −1.4 to 1.7, whereas the *outfit* was ranging from −1.4 to 1.5.

The acceptable values for item polarity or point measure correlation (PTMEA) should be more than 0.30. As shown in Table 5 for the item polarity of each variable, the values across the constructs are ranging from 0.53 to 0.77. The raw variance explained by the measures represents more than 40% of the raw variance explained by each variable, while the Eigenvalue is less than 5.0 to prove the unidimensionality of the items. The noise of the items is also less than 15%, and this is likewise acceptable for the items to be proven unidimensional. Additionally, as can be seen in Table 6, which shows the item local independence of each variable, the correlation between the pair of items is not more than 0.7 and, hence, is acceptable for the quality of measurement.

Based on the analysis, 21 out of the 79 items analysed were found to be misfit. After conducting in-depth research on these items, the researchers decided to drop all 21 problematic items after considering and cross-checking the experts’ comments based on the content validity of these 21 items. Moreover, further analysis was conducted using the Rasch measurement model after removing these items to obtain better analytical findings from the remaining 58 items, which had accordingly achieved the set values of item fit, polarity, local independence, and unidimensional characteristics. Besides, in terms of the reliability and separation index values as shown in Table 7, Table 8, Table 9, Table 10, Table 11 and Table 12, the Rasch measurement model analysis showed good results for each scale of teachers’ efficacy, school leaders’ transformational leadership practices, and teachers’ innovative behaviour.

Overall, the values reported in each statistical summary of items across the three variables of teachers’ efficacy, school leaders’ transformational leadership practices, and teachers’ innovative behaviour are acceptable. For the teachers’ self-efficacy scale, the reliability index value of the items was good and exceeded the recommended value with 0.81, while the separation index value was also good and exceeded the recommended value with 2.09. As for the school leaders’ transformational leadership scale, the reliability index value of the items and the separation index value likewise exceeded the recommended values with 0.83 and 2.19, respectively. Finally, for the teachers’ innovative behaviour scale, the reliability index value of the items was good and exceeded the recommended value with 0.80, while the separation index value was also good and exceeded the recommended value with 2.02. Additionally, the values reported in each statistical summary of individuals (persons) across the three variables were accepted and sufficient for measurement with more than 2.00 for separation index and more than 0.8 for person reliability.

Table 13 shows the consistency reliability values based on Cronbach’s alpha coefficient and the analysis showed relatively good results for each of the constructs in each scale of teachers’ efficacy, teachers’ innovative behaviour, and school leaders’ transformational leadership practices. For the teachers’ self-efficacy scale, the Cronbach’s alpha values for all three constructs were between 0.784 to 0.866, while four constructs in the teachers’ innovative behaviour scale were ranging from 0.788 to 0.56 and four other constructs in the school leaders’ transformational leadership scale were between 0.802 and 0.839.

Table 14 shows the total items dropped in pilot testing. Out of 79 items, 21 items were dropped for not fulfilling the item fit requirement in Rasch; however, no items were dropped during the stage of analysis for item polarity and local independence. Finally, 58 items remained, with 24 items for self-efficacy, 30 items for innovative behaviour, and 25 items for transformational leadership to be applied in the actual study.

#### 3.2.2. Sociodemographic Characteristics of Respondents

The sociodemographic characteristics of the respondents include eight items on the respondents’ general background such as age, gender, ethnicity, job position, school type, school location, teaching experience, and academic qualification. For instance, in the age section, the age range of the respondents is between 20 and 60 years old, while the job position section consists of regular teachers or administrators. As for the type of school, only two schools were involved in the study, namely primary and secondary schools, whereas information on the school location includes either urban or rural areas.

#### 3.2.3. Teachers’ Innovative Behaviour Scale

This section consists of 20 items for measuring teachers’ innovative behaviour based on four constructs, namely identifying or exploring opportunities (5 items), generating ideas (5 items), promoting ideas (5 items), and realising ideas (5 items). This section was also based on the innovative behaviour measurement developed by [12,13], combined with and modified based on the previous studies by [64,65,66] to ensure its suitability in the local context. Additionally, a 5-point Likert scale was further used in the innovative behaviour measurement scale, which ranges from 1 (Never) to 5 (Very Frequent).

#### 3.2.4. Teachers’ Efficacy Scale

This section comprises a total of 18 items for measuring teachers’ self-efficacy, which was divided into three constructs that include 6 items for each construct of student involvement (teachers’ confidence in involving students in the learning process), teaching strategy (teachers’ confidence in making students understand), and classroom management (teachers’ confidence in controlling the class). This section was also adapted from the studies by [61,62,70], and the items were measured using a 5-point Likert scale that ranges from 1 (Not Confident at All) to 5 (Very Confident).

#### 3.2.5. School Leaders’ Transformational Leadership Scale

This section measures the variable of school leaders’ transformational leadership practices in which the instruments used by [67,68] were adapted and modified by the researcher to generate the items for this variable. Overall, this section consists of 20 items based on four constructs, namely individualised consideration (5 items), intellectual stimulation (5 items), inspirational motivation (5 items), and idealised influence (5 items). A 5-point Likert scale that ranges from 1 (Highly Disagree) to 5 (Highly Agree) was also used to measure the items in the school leaders’ transformational leadership scale.

### 3.3. Data Analysis

This study involved two types of data analyses, namely descriptive and inferential analyses. The gathered data were analysed using the Statistical Package for Social Sciences (SPSS) software version 26.0 (IBM Corporation, New York, NY, USA), while the Enter method was used in the regression analysis to identify the relative influence of the independent variables on the dependent variable.

Before conducting the analyses, data checking and cleaning were initially conducted to ensure that the data were free from errors. The data also went through a checking process using SPSS descriptive statistics to detect any missing data and wrongly entered data. Information such as frequency, minimum and maximum values, mean, median, and mode from SPSS descriptive statistics were further examined to detect any errors.

## 4. Results

In the actual study, a total of 1500 questionnaires were distributed to the respondents and 1415 questionnaires were returned to the researchers, giving a recovery rate of 94.33%. The research results are explained as follows:

### The influence of Teachers’ Self-Efficacy and School Leaders’ Transformational Leadership Practices on Teachers’ Innovative Behaviour

Multiple regression analysis was used to assess the probability of the significant influence of teachers’ self-efficacy and school leaders’ transformational leadership practices (independent variables) on teachers’ innovative behaviour (dependent variable). Table 15 and Table 16 show the multiple regression analysis results.

Based on the analysis, the regression model was significant (*F*(2,1412) = 627.60, *p* < 0.001, and *R*^2^ = 0.470) and both factors of teachers’ self-efficacy (*b* = 0.781, *t* = 25.501, *p* < 0.001) and school leaders’ transformational leadership practices (*b* = 0.235, *t* = 9.887, *p* < 0.001) in this study were significant predictors of teachers’ innovative behaviour.

The regression analysis results also revealed that, overall, teachers’ self-efficacy and school leaders’ transformational leadership practices could explain 47.0% of the variance in teachers’ innovative behaviour (*R*^2^ = 0.470), while the remaining 53.0% of the variance was explained by other factors that were not examined in this study.

As suggested by [71,72] on the relative contribution of each independent variable, a reference was made to the weighted Beta value, which is better known as the standardised coefficient value. The standardised coefficient value was used to compare the contribution of each independent variable with the highest standardised coefficient indicating that the variable had a more dominant effect or contribution [72].

Based on Table 17, the standardised coefficient value of teachers’ self-efficacy (0.558) was higher than the standardised coefficient value of school leaders’ transformational leadership practices (0.216). This shows that teachers’ self-efficacy had a higher effect on teachers’ innovative behaviour than school leaders’ transformational leadership practices.

Overall, the contribution of the two independent variables of teachers’ self-efficacy and school leaders’ transformational leadership practices to the variation of change in teachers’ innovative behaviour can be shaped through a regression equation model. Based on the findings of the analysis obtained in Table 16, the regression equation model for this study can be formed as follows:

Teachers’ Innovative Behaviour = −2.306 + (0.781) Teachers’ Self-Efficacy + (0.235) School Leaders’ Transformational Leadership Practices

From this equation, it can be summarised that every increase in one unit of teachers’ self-efficacy will also increase the innovative behaviour of teachers by 0.781 units, while every addition to one unit of school leaders’ transformational leadership practices will further increase teachers’ innovative behaviour by 0.235 units.

## 5. Discussion

The findings showed that both independent variables of teachers’ self-efficacy and school administrators’ (principals/headmasters/headmistresses) transformational leadership practices had a significant influence on teachers’ innovative behaviour.

Specifically, based on the multiple regression analysis results, 47% of the changes in the score of teachers’ innovative behaviour could be explained by the school administrators’ transformational leadership practices and teachers’ self-efficacy, while the remaining 53% was contributed by other factors. However, even with only 47%, this value is good enough and adequate for research in the social sciences field because the value exceeds 0.30% or 30% [73]. Therefore, this value can represent the importance or influence of the two variables on teachers’ innovative behaviour.

In general, the findings showed that the factors of self-efficacy and transformational leadership practices both require attention due to their huge influence on teachers’ innovative behaviour. Besides, the significant contribution to the variability in teachers’ innovative work behaviour reflects the importance of both variables in developing the innovative behaviour of teachers because both of these factors have distinctive roles in terms of personal and environmental factors in influencing innovative behaviour.

In the context of this study, self-efficacy is undeniably essential in influencing teachers’ innovative behaviour, and this can be explained in several ways. Firstly, individuals with high self-efficacy levels are more inclined to perform challenging and risky tasks as well as exerting more effort to ensure that the tasks can be completed properly compared to those with low self-efficacy levels [36]. This is because individuals with high self-efficacy have higher motivation, belief, and confidence in their own capabilities. Therefore, when teachers have high self-efficacy levels, they are indirectly more confident in their knowledge and skills to participate in challenging tasks such as innovative behaviour [41].

Besides, teachers with high self-efficacy are also motivated to achieve positive results by spending more time to identify problems, generate new ideas, and consequently implement the ideas to solve problems [74,75]. Reference [76] added that teachers with high self-efficacy are more open and show a higher exuberance and desire to think of ideas and try new things, which indirectly encourages them to be involved in innovative behaviour.

Compared to teachers with low self-efficacy, teachers with high self-efficacy will also be more prepared to face any challenges and uncertainties because they tend to see challenges as opportunities and endure through difficulties [77,78,79]. As discussed in existing literature, it is difficult to possess innovative behaviour because it involves risks and uncertainties that may end with failure. Therefore, teachers with high self-efficacy levels are indirectly more prepared to take a risk through innovative behaviour because they may see challenges in their tasks as opportunities and may not feel daunted to expand or instil new ideas in executing their tasks despite the risk of failure.

In the same vein, school administrators’ (principals/headmasters/headmistresses) transformational leadership practices are also a significant factor that influences and contributes to innovative behaviour among teachers. In line with the transformational leadership theory expounded by [49], effective and positive leadership practices such as transformational leadership are necessary to influence followers to demonstrate positive behaviour. Hence, as leaders in the school organisation, the principals/headmasters/headmistresses play an important role in influencing innovative behaviour among teachers.

Besides, in the context of this study, the influence of school administrators’ transformational leadership practices on teachers’ innovative behaviour can be explained by the concept and dimensions of these leadership practices, which may contribute to teachers’ innovative behaviour through a series of behaviours such as encouraging learning activities, information sharing, and intellectual stimulation.

From the perspective of the theory explicated by [49], the leadership practices among school administrators should provide strong support to teachers’ innovative behaviour. This is because the dimensions in these leadership practices are capable of stimulating teachers’ thinking and imagination, encouraging teachers to be more creative and innovative, and training teachers to think strategically [80,81]. Specifically, principals/headmasters/headmistresses who practise the transformational leadership style will always encourage teachers to think outside the box, especially when facing challenges and looking for new methods to implement and solve problems in their tasks by using intellectual stimulation. Such stimulation indirectly improves teachers’ intellectual capacity and exploratory thinking while encouraging them to be more creative in producing new and original ideas that will certainly encourage them to have innovative behaviour [55,82,83,84,85,86].

Additionally,, individualised consideration practised by the school administrators in transformational leadership also demonstrates consideration, empathy, and individual support to teachers, which will indirectly help them build their confidence and result in higher levels of creativity and innovation [87]. As such, the ability of the principals/headmasters/headmistresses to consider differences in terms of the strengths, weaknesses, and needs of teachers would help the teachers realise and fully utilise their capabilities for more effective task executions that consequently leads to innovative behaviour [82,88]

Finally, innovative behaviour among teachers may also be contributed by idealised influence and inspirational motivation practised by school administrators. For instance, the principals/headmasters/headmistresses may use these two aspects to highly motivate teachers, especially in executing tasks. As a result, the teachers’ confidence and creativity will increase and this further stimulates their innovative behaviour [84,89,90].

## 6. Limitations

Since the results of this study must be interpreted with caution, several research limitations should be borne in mind before analysing any possible directions for future research. Firstly, this study was conducted using cross-sectional data that were solely based on questionnaires; hence, it was difficult to review the causal relationships between variables. Although the quantitative data used in this study had revealed each relationship, the data may not be able to explain why such an association exists [91]. However, this issue can be overcome by collecting longitudinal data to provide more applicable findings as well as using qualitative data to further identify the relationships between variables. Likewise, a mixed method (qualitative and quantitative data) can also provide broader findings on the relationships between teachers’ self-efficacy and school administrators’ transformational leadership practices with teachers’ innovative behaviour.

Secondly, since the data in this study were only collected from school teachers in two types of schools in Malaysia, namely primary school or *Sekolah Kebangsaan* (SK) and secondary school or *Sekolah Menengah Kebangsaan* (SMK), it is rather improper to generalise the findings of this study to all teachers. Thus, it is suggested that future research includes a bigger scope within the national and international contexts with a greater sample size to generalise the results and observe the consistency of the current findings. It would also be more exciting to analyse and compare the current findings to see whether or not they are reproducible in other cultures and backgrounds different from this study.

Thirdly, this research was only focusing on the Rasch analysis to determine the validity and reliability of the item and the subscale. Rasch analysis is an advanced approach fitting those able focuses on analysis until at the item level compared to the Confirmatory Factor Analysis (CFA) or any other similar analysis that puts emphasis on verifying the factor structure of a set of observed variables. Therefore, Rasch analysis considers the person’s ability and the item difficulty to assess the psychometric property by allowing estimation of item difficulty parameters that are “sample-independent” because their values do not depend on traits or attitudes distribution across samples. For future research, we strongly suggest to expand the item assessment using Confirmatory Factor Analysis (CFA) to provide more relevant information from classical test theory perspectives for construct validity assessment. Therefore, we can barely look in more depth at the factors underlying the study variables and investigate how well the measured variables in the instrument represent the number of constructs in this study.

## 7. Conclusions

This study proposed and tested a hypothesis to explain the relationships between teachers’ self-efficacy, and school leaders’ transformational leadership practices, towards teachers’ innovative behaviour. The findings that have been presented confirmed that both teachers’ self-efficacy and school leaders’ transformational leadership practices are the factors influencing teachers’ innovative behaviour, which could explain 47.0% of the total variance. It is important for the Ministry of Education to strengthened the content of self-efficacy and transformational leadership practices in the innovative behaviour training among teachers. The findings are important for both factors to play an important role in influencing the innovative behaviour among teachers. As a result of conducting this research, this study proposed drafting policies on related programmes to improve teachers’ innovative behaviour. If stakeholders and policymakers were to take this study seriously, they might consider the aspects of teachers’ self-efficacy and transformational leadership practices by school administrators such as the principals/headmasters/headmistresses, especially for self-career development. This is to ensure that teachers are equipped with innovative behaviour to ensure that our teachers are still relevant in the education system. Additionally, this will indirectly be encouraging the teachers to face challenges and obstacles in a rapidly changing open environment.

## Figures and Tables

**Table 1:** Item evaluation for expert consensus.

CVR ^1^ Value	Total Items			Action
	Innovative Behaviour	Self-Efficacy	Transformational Leadership	
1.000	12	20	15	Accepted
0.818	13	2	4	Accepted
0.636	2	2	3	Accepted
<0.636	7	4	3	Re-evaluated
Dropped	4	4	0	-
Final items	30	24	25	79 items

^1^ Content Validity Ratio.

**Table 2:** Item fit for self-efficacy items.

Item Code	Total Score	Logits	S.E.	INFIT		OUTFIT	
				MNSQ ^1^	ZSTD ^2^	MNSQ ^1^	ZSTD ^2^
PM1	448	−0.30	0.20	1.25	1.50	1.06	0.40
* PM2	441	−0.03	0.19	1.37	2.20	1.20	1.20
* PM3	441	−0.03	0.19	0.69	−2.10	0.69	−2.00
PM4	452	−0.46	0.20	0.96	−0.20	0.96	−0.20
* PM5	442	−0.07	0.19	0.70	−2.00	0.68	−2.00
PM6	449	−0.34	0.20	1.08	0.60	1.12	0.70
PM7	449	−0.34	0.20	1.10	0.70	1.10	0.60
* PM8	445	−0.18	0.20	0.73	−1.80	0.70	−1.90
PM9	450	−0.38	0.20	0.92	−0.40	0.91	−0.50
PM10	444	−0.14	0.20	1.06	0.40	1.03	0.20
SP1	450	−0.38	0.20	1.28	1.70	1.17	1.00
SP2	440	0.01	0.19	1.16	1.00	1.26	1.50
SP3	453	−0.50	0.20	1.05	0.40	0.94	−0.30
SP4	449	−0.34	0.20	0.94	−0.30	0.86	−0.80
SP5	443	−0.10	0.20	1.19	1.20	1.05	0.40
SP6	436	0.16	0.19	0.86	−0.90	0.95	−0.20
* BD1	425	0.55	0.18	0.81	−1.20	0.71	−1.90
BD2	447	−0.26	0.20	1.27	1.70	1.09	0.60
* BD3	449	−0.34	0.20	0.81	−1.30	0.73	−1.70
BD4	430	0.37	0.19	0.88	−0.80	0.83	−1.10
BD5	419	0.75	0.18	0.97	−0.10	0.86	−0.90
BD6	415	0.88	0.18	1.02	0.20	0.98	−0.10
BD7	427	0.48	0.19	1.03	0.30	1.11	0.70
BD8	412	0.97	0.18	0.95	−0.30	1.00	0.10

^1^ Mean Square; ^2^ Z-score Standardized; * Item that did not fit. References: Self-efficacy—PM: student involvement; BD: classroom management; SP: teaching strategy.

**Table 3:** Item fit for innovative behaviour items.

Item Code	Total Score	Logits	S.E.	INFIT		OUTFIT	
				MNSQ ^1^	ZSTD ^2^	MNSQ ^1^	ZSTD ^2^
IE1	416	−0.07	0.17	1.14	1.0	1.11	0.8
IE2	416	−0.07	0.17	0.90	−0.7	0.85	−1.1
* IE3	419	−0.16	0.17	0.80	−1.5	0.74	−1.9
* IE4	391	0.64	0.16	1.33	2.2	1.45	2.8
IE5	390	0.67	0.16	0.90	−0.6	0.91	−0.6
* IE6	409	0.14	0.17	1.49	3.1	1.42	2.6
IE7	406	0.22	0.17	1.20	1.4	1.17	1.2
IE8	431	−0.53	0.18	0.80	−1.4	0.80	−1.4
IG1	419	−0.16	0.17	0.94	−0.4	0.92	−0.5
* IG2	328	2.16	0.15	0.59	−3.5	0.64	−2.9
* IG3	430	−0.50	0.18	0.72	−2.2	0.66	−2.6
IG4	425	−0.34	0.18	0.88	−0.9	0.81	−1.4
IG5	450	−1.14	0.18	0.87	−0.9	0.82	−1.2
IG6	435	−0.65	0.18	1.02	0.2	0.98	−0.1
* IG7	400	0.39	0.17	1.52	3.2	1.56	3.4
IG8	420	−0.19	0.17	1.11	0.8	1.08	0.6
IP1	427	−0.4	0.18	0.86	−1.0	0.85	−1.0
IP2	416	−0.07	0.17	0.86	−1.0	0.82	−1.3
* IP3	430	−0.50	0.18	0.74	−2.0	0.69	−2.3
* IP4	331	2.10	0.15	0.63	−3.1	0.66	−2.8
IP5	404	0.28	0.17	1.11	0.8	1.08	0.6
IP6	419	−0.16	0.17	1.22	1.5	1.12	0.8
IP7	419	−0.16	0.17	1.11	0.8	1.02	0.2
II1	418	−0.13	0.17	1.16	1.1	1.14	1.0
* II2	435	−0.65	0.18	1.35	2.3	1.29	1.8
II3	436	−0.69	0.18	0.87	−0.9	0.79	−1.4
II4	437	−0.72	0.18	1.10	0.8	1.02	0.2
II5	405	0.25	0.17	0.87	−0.9	0.87	−0.9
* II6	400	0.39	0.17	1.54	3.3	1.54	3.3
II7	412	0.05	0.17	0.87	−0.9	0.89	−0.7

^1^ Mean Square; ^2^ Z-score Standardized; * Item that did not fit. References: Innovative behaviour—IP: promoting ideas; II: realising ideas; IE: exploring opportunities; IG: generating ideas.

**Table 4:** Item fit for transformational leadership items.

Item Code	Total Score	Logits	S.E.	INFIT		OUTFIT	
				MNSQ ^1^	ZSTD ^2^	MNSQ ^1^	ZSTD ^2^
IC1	416	0.14	0.17	1.07	0.50	1.16	1.00
* IC2	419	0.05	0.17	0.75	−1.70	0.69	−2.10
IC3	414	0.20	0.17	0.93	−0.40	0.94	−0.30
IC4	398	0.64	0.16	1.15	1.00	1.25	1.50
IC5	390	0.84	0.16	0.81	−1.30	0.84	−1.00
* IC6	435	−0.45	0.18	1.43	2.50	1.39	2.10
IC7	431	−0.32	0.18	0.81	−1.20	0.84	−1.00
* IC8	409	0.34	0.17	1.42	2.50	1.37	2.10
IS1	419	0.05	0.17	0.87	−0.80	0.93	−0.40
IS2	416	0.14	0.17	0.82	−1.20	0.82	−1.10
IS3	418	0.08	0.17	1.10	0.70	1.18	1.10
IS4	425	−0.13	0.18	0.90	−0.60	0.83	−1.00
IS5	406	0.42	0.17	1.12	0.80	1.14	0.90
* IM1	430	−0.29	0.18	0.78	−1.50	0.73	−1.80
IM2	420	0.02	0.17	1.13	0.90	1.13	0.90
IM3	427	−0.19	0.18	0.83	−1.10	0.86	−0.80
IM4	419	0.05	0.17	1.14	0.90	1.04	0.30
IM5	404	0.48	0.16	0.99	0.00	1.14	0.90
IM6	419	0.05	0.17	1.11	0.80	0.99	0.00
II1	437	−0.51	0.18	1.14	0.90	1.04	0.30
* II2	430	−0.29	0.18	0.71	−2.00	0.66	−2.30
II3	436	−0.48	0.18	0.90	−0.60	0.82	−1.00
II4	447	−0.85	0.19	1.10	0.70	0.96	−0.10
II5	435	−0.45	0.18	1.08	0.60	1.06	0.40
II6	405	0.45	0.16	0.83	−1.10	0.89	−0.70

^1^ Mean Square; ^2^ Z-score Standardized; * Item that did not fit. References: Transformational leadership—IC: individualised consideration; IS: intellectual stimulation; II: idealised influence; IM: inspirational motivation.

**Table 5:** Unidimensionality and item polarity of each variable.

Variable	Unidimensionality Inspection			PTMEA ^1^ Range	
	Raw Variance Explained by Measures (>40%)	Eigen Value(<5.0)	Noise (<15%)	Minimum Value	Maximum Value
Self-efficacy	41.2%	3.4	11.0%	0.54	0.69
Innovative behaviour	46.1%	2.8	7.5%	0.55	0.77
Transformational leadership	44.3%	3.1	8.7%	0.53	0.73

^1^ Point Measure Correlation.

**Table 6:** Item local independence of each variable.

**Correlation for Self-Efficacy**	**Item Pair**	**Correlation for** **Innovative Behaviour**	**Item Pair**	**Correlation for Transformational Leadership**	**Item Pair**
0.32	PM1–PM4	0.55	IP2–II7	0.43	IC1–IC3
0.31	BD5–BD8	0.46	IE5–IE7	0.42	IC3–IC4
0.31	SP6–BD5	0.43	IE1–IE2	0.42	IC5–IS5
−0.43	SP3–BD5	0.42	IP2–II1	0.40	II5–II6
−0.38	PM1–BD8	0.41	IE2–IE7	0.39	IS2–IS3
−0.36	PM9–SP5	0.37	IG6–II5	0.38	IC3–IS5
−0.35	PM1–BD7	0.36	IE1–IE7	−0.40	IC4–II4
−0.35	SP3–BD2	0.35	IE1–IE5	−0.35	IM4–II4
−0.33	PM4–BD8	0.34	IG5–IG6	−0.34	IM4–II5
−0.33	SP1–BD5	−0.41	IG5–IP6	−0.33	IC5–IM5

References: Self-efficacy—PM: student involvement; BD: classroom management; SP: teaching strategy. Innovative behaviour—IP: promoting ideas; II: realising ideas; IE: exploring opportunities; IG: generating ideas. Transformational leadership—IC: individualised consideration; IS: intellectual stimulation; II: idealised influence; IM: inspirational motivation.

**Table 15:** Regression model summary.

	*R*	*R^2^*	Adjusted *R*^2^
Model 1	0.686	0.471	0.470

**Table 16:** Variance analysis of the regression model.

	Sum of Squares	df	Mean Square	*F*	*p*-Value
Regression	71,030.01	2	35,515.00	627.60	0.000
Residual	79,902.63	1412	56.59		
Total	150,932.64	1414			

**Table 17:** Multiple regression coefficient analysis.

Variable	Non-Standardised Coefficient		Standardised Coefficient	*t*	*p*-Value
	B	S.E.	Beta		
Constant	−2.306	2.182		−1.057	0.291
Teachers’ Self-efficacy	0.781	0.031	0.558	25.501	0.000
School Leaders’ Transformational Leadership	0.235	0.024	0.216	9.887	0.000

**Table 7:** Statistical summary of items (self-efficacy).

Statistics	Raw Score	Count	Measure	Model Error	Infit		Outfit	
					MNSQ ^1^	ZSTD ^2^	MNSQ ^1^	ZSTD ^2^
Mean	439.6	109.0	0.01	0.19	1.05	0.4	1.01	0.1
Standard Deviation	13.0	0.0	0.47	0.01	0.13	0.8	0.11	0.7
Max.	453.0	109.0	0.97	0.20	1.28	1.7	1.26	1.5
Min.	412.0	109.0	−0.50	0.18	0.86	−0.9	0.83	−1.1
Real RMSE ^3^	0.20	Adj. SD ^4^	0.42	Separation	2.09	Item Reliability		0.81
Model RMSE ^3^	0.19	Adj. SD ^4^	0.42	Separation	2.20	Item Reliability		0.83

^1^ Mean Square; ^2^ Z-score Standardized; ^3^ Root Mean-Square Error; ^4^ Adjusted Standard Deviation.

**Table 8:** Statistical summary of individuals (self-efficacy).

Statistics	Raw Score	Count	Measure	Model Error	Infit		Outfit	
					MNSQ ^1^	ZSTD ^2^	MNSQ ^1^	ZSTD ^2^
Mean	96.8	24.0	2.63	0.46	0.96	−0.5	0.96	−0.5
Standard Deviation	10.4	0.0	1.97	0.25	0.74	2.1	0.75	2.2
Max.	120.0	24.0	8.85	1.83	3.33	4.2	3.37	4.4
Min.	73.0	24.0	−0.67	0.29	0.04	−4.8	0.04	−4.8
Real RMSE ^3^	0.56	Adj. SD ^4^	1.89	Separation	3.37	Person Reliability		0.92
Model RMSE ^3^	0.52	Adj. SD ^4^	1.90	Separation	3.63	Person Reliability		0.93

^1^ Mean Square; ^2^ Z-score Standardized; ^3^ Root Mean-Square Error; ^4^ Adjusted Standard Deviation.

**Table 9:** Statistical summary of items (innovative behaviour).

Statistics	Raw Score	Count	Measure	Model Error	Infit		Outfit	
					MNSQ ^1^	ZSTD ^2^	MNSQ ^1^	ZSTD ^2^
Mean	420.1	109.0	−0.20	0.17	0.99	−0.1	0.95	−0.3
Standard Deviation	13.3	0.0	0.40	0.00	0.13	0.9	0.13	0.9
Max.	450.0	109.0	0.67	0.18	1.22	1.5	1.17	1.2
Min.	390.0	109.0	−1.14	0.16	0.80	−1.4	0.79	−1.4
Real RMSE ^3^	0.18	Adj. SD ^4^	0.36	Separation	2.02	Item Reliability		0.80
Model RMSE ^3^	0.17	Adj. SD ^4^	0.36	Separation	2.09	Item Reliability		0.81

^1^ Mean Square; ^2^ Z-score Standardized; ^3^ Root Mean-Square Error; ^4^ Adjusted Standard Deviation.

**Table 10:** Statistical summary of individuals (innovative behaviour).

Statistics	Raw Score	Count	Measure	Model Error	Infit		Outfit	
					MNSQ ^1^	ZSTD ^2^	MNSQ ^1^	ZSTD ^2^
Mean	113.5	30.0	1.83	0.33	0.99	−0.3	0.99	−0.3
Standard Deviation	14	0.0	1.51	0.04	0.62	2.2	0.64	2.2
Max.	147.0	30.0	6.55	0.63	2.89	5.0	2.98	5.2
Min.	66.0	30.0	−2.14	0.25	0.13	−4.8	0.13	−4.9
Real RMSE ^3^	0.37	Adj. SD ^4^	1.47	Separation	3.97	Person Reliability		0.94
Model RMSE ^3^	0.33	Adj. SD ^4^	1.48	Separation	4.41	Person Reliability		0.95

^1^ Mean Square; ^2^ Z-score Standardized; ^3^ Root Mean-Square Error; ^4^ Adjusted Standard Deviation.

**Table 11:** Statistical summary of items (transformational leadership).

Statistics	Raw Score	Count	Measure	Model Error	Infit		Outfit	
					MNSQ ^1^	ZSTD ^2^	MNSQ ^1^	ZSTD ^2^
Mean	439.6	109.0	0.01	0.19	1.05	0.4	1.01	0.1
Standard Deviation	13.0	0.0	0.47	0.01	0.13	0.8	0.11	0.7
Max.	453.0	109.0	0.97	0.20	1.28	1.7	1.26	1.5
Min.	412.0	109.0	−0.50	0.18	0.86	−0.9	0.83	−1.1
Real RMSE ^3^	0.20	Adj. SD ^4^	0.42	Separation	2.09	Item Reliability		0.81
Model RMSE ^3^	0.19	Adj. SD ^4^	0.42	Separation	2.20	Item Reliability		0.83

^1^ Mean Square; ^2^ Z-score Standardized; ^3^ Root Mean-Square Error; ^4^ Adjusted Standard Deviation.

**Table 12:** Statistical summary of individuals (transformational leadership).

Statistics	Raw Score	Count	Measure	Model Error	Infit		Outfit	
					MNSQ ^1^	ZSTD ^2^	MNSQ ^1^	ZSTD ^2^
Mean	96.8	24.0	2.63	0.46	0.96	−0.5	0.96	−0.5
Standard Deviation	10.4	0.0	1.97	0.25	0.74	2.1	0.75	2.2
Max.	120.0	24.0	8.85	1.83	3.33	4.2	3.37	4.4
Min.	73.0	24.0	−0.67	0.29	0.04	−4.8	0.04	−4.8
Real RMSE ^3^	0.56	Adj. SD ^4^	1.89	Separation	3.37	Person Reliability		0.92
Model RMSE ^3^	0.52	Adj. SD ^4^	1.90	Separation	3.63	Person Reliability		0.93

^1^ Mean Square; ^2^ Z-score Standardized; ^3^ Root Mean-Square Error; ^4^ Adjusted Standard Deviation.

**Table 13:** Cronbach’s alpha coefficient by construct for all variables.

Variable Construct	Cronbach’s Alpha Coefficient
Teachers’ self-efficacy	
Student involvement	0.866
Teaching strategy	0.784
Classroom management	0.860
Teachers’ innovative behaviour	
Exploring opportunities	0.845
Generating ideas	0.793
Promoting ideas	0.856
Realising ideas	0.788
School leaders’ transformational leadership	
Individualised consideration	0.839
Intellectual stimulation	0.818
Inspirational motivation	0.803
Idealised influence	0.829

**Table 14:** Total items dropped in pilot testing.

Variable	Pilot Test Items	Total Items Dropped			Remaining Items
		Item Fit	Item Polarity	Local Independence	
Self-efficacy	24	6	-	-	18
Innovative behaviour	30	10	-	-	20
Transformational leadership	25	5	-	-	20
Total	79	21	0	0	58

## Data Availability

The data presented in this study are available on request from the corresponding author. The data are not publicly available due to the risk of identification of study participants.

## Appendix A

Teachers’ innovative behaviour scale items [12,13,59,60,61]

1. Identify the needs of my clients (students, parents, community).

2. Identify opportunities that can be taken.

3. Looking for opportunities to advance the organisation.

4. Think about producing innovations that can be used to achieve organisational goals.

5. Think about improvements in my work.

6. Generate new ideas.

7. Submit innovative ideas.

8. Give suggestions for improvement on the ideas given.

9. Find a solution to a problem using new techniques/methods.

10. Find new approaches to accomplish tasks.

11. Actively engage in promoting new ideas to colleagues.

12. Actively engage in promoting new ideas to superiors.

13. Actively engage in informing others about the progress of a new idea.

14. Convince others of the importance of a new idea.

15. Actively engage in highlighting new ideas so that they have the opportunity to be implemented.

16. Strive to develop something new.

17. Analyse the undesirable effects when new ideas are put into practice.

18. Realise a new idea into something useful.

19. Introduce new ideas in the organisation systematically.

20. Contribute energy to realise new ideas.

Teachers’ efficacy scale items [61,62,63]

1. Confidence to control students’ problematic behaviour in class.

2. Confidence to ensure students obey class rules.

3. Confidence to take appropriate action against troubled students.

4. Confidence to prevent troubled students from interrupting the entire PdPc session.

5. Confidence to manage student discipline in the classroom without stress.

6. Confidence to ensure that students pay attention throughout the PdPc session.

7. Confidence to help students think critically.

8. Confidence to motivate students who have low interest in school assignments.

9. Confidence to make students believe that they can succeed in school assignments.

10. Confidence to instil student creativity.

11. Confidence to improve the performance of weak students.

12. Confidence to ensure students’ participation in activities during PdPc.

13. Confidence to measure students’ understanding of what has been taught.

14. Confidence to change the PdPc method based on the appropriate level for each student.

15. Confidence to use a variety of evaluation strategies.

16. Confidence to give alternative explanations when students feel confused.

17. Confidence to provide appropriate needs for intelligent students.

18. Confidence to diversify teaching techniques.

School leaders’ transformational leadership scale items [67,68]

1. My school administrators help develop my personal potential.

2. My school administrators treat me as an individual rather than just as a member of a group.

3. My school administrators know what I want.

4. My school administrators took their time to guide me.

5. My school administrators know the talents of each teacher.

6. My school administrators emphasise the use of my discretion to solve problems.

7. My school administrators seek various perspectives when solving a problem.

8. My school administrators make me see a problem from various angles.

9. My school administrators suggest some new ways to complete projects.

10. My school administrators explain the importance of having a clear purpose.

11. My school administrators are always together in carrying out tasks.

12. My school administrators emphasise the importance of having a shared mission.

13. My school administrators express the future with an optimistic outlook.

14. My school administrators state the things that need to be done with full enthusiasm.

15. My school administrators set high standards for each job.

16. My school administrators state with confidence that the goal can be achieved.

17. My school administrators place high expectations on the ability of teachers.

18. My school administrators have a clear mission and vision in achieving organisational goals.

19. My school administrators display the characteristics of self-confidence in decision making.

20. My school administrators get full confidence from all teachers.
